# A neuropsychiatric complication of oligomenorrhea according to iranian traditional medicine

**Published:** 2014-07

**Authors:** Maryam Yavari, Faezeh Khodabandeh, Mojgan Tansaz, Safoura Rouholamin

**Affiliations:** 1 *Department of Traditional Medicine, School of Traditional Medicine, Shahid Beheshti University of Medical Sciences, Tehran, Iran.*; 2 *Isfahan University of Medical Sciences, Isfahan, Iran.*; 3 *Department of Obstetrics and Gynecology, School of Medicine, Isfahan University of Medical Sciences, Isfahan, Iran.*

**Keywords:** *Iranian traditional medicine*, *Oligomenorrhea*, *Uterine strangulation*, *Menstruation Disturbances*

## Abstract

Oligomenorrhea, a prevalent disease with serious complications, has been declared in the Avicenna traditional medicine in detail. Avicenna in his famous book, Cannon of Medicine, presents a syndrome termed ‘uterine strangulation’, as a complication of menstrual bleeding cessation and lack of sexual satisfaction. We have explained this syndrome from both traditional and conventional medicine viewpoints to propose a new hypothesis for diagnosis and treatment of women with oligomenorrhea and systemic signs/symptoms admitting to clinics for further evaluation. This hypothesis definitely needs to be further assessed and confirmed by strong clinical trials.

## Introduction

Oligomenorrhea with a prevalence of 12-15.3% in different studies around the world, is one of the most common types of menstrual bleeding disorders (1-7). In recent decades, as a result of changes in life style, obesity, low physical activity, unhealthy nutrition, and emotional stress, the prevalence of amenorrhea and oligomenorrhea has increased considerably (8). Among several etiologic factors, polycystic ovarian disease (PCOD) is the most important underlying factor for oligomenorrhea (9, 10). According to conventional medicine, negligence to treat menstrual bleeding cessation can lead to several complications-especially in PCO patients- that include low fertility, lowering bone density, endometrial and breast cancer, coronary and brain artery disease, diabetes, hirsutism and acnea (11-13).

Some studies have even explained emotional complications of oligomenorrhea and its effect on economy and social productivity (14-17). In Iranian traditional medicine (ITM), the term “Ehtebas Tams” is used equivalent to the terms oligomenorrhea, amennorhea and hypomenorrhea in the conventional medicine (18). According to ITM, menstrual bleeding in its normal quality and quantity, guarantees the health of women in reproductive age and results in controlling excessive erotic emotions (18-21). Menstruation is an important excretory pathway therefore the cessation of bleeding results in spreading the excreta material through the whole body and developing systemic signs/symptoms (18-21).

One of the complications mentioned in the ITM textbooks for oligomenorrhea is a syndrome called “Ekhtenagh rahem” which may be translated to “uterine strangulation” (22). This is a disease of uterine origin; due to the inter-organ relationships mentioned in the ITM, the symptoms are mostly neuropsychiatric and are similar to a seizure or faint attack though (19-21). The authors are going to explain this syndrome in detail and then discuss possible mechanisms and disorders that are in accordance with this syndrome from conventional medicine viewpoint.

**Physiopathology** **of the uterine strangulation**

Avicenna (980-1037 A.D.), the most famous physician of the ITM (also known as humoral system of medicine) in his famous book “Canon”, describes that following menstrual bleeding cessation or lack of sexual satisfaction, the excreta accumulates in the uterus (21). There are some inter organ relationships mentioned in the ITM textbooks, one is the relationship of the uterus organ with brain and heart (19-21). Due to this links, sometimes the origin of a disease is in one organ while the signs/symptoms appear in a second organ (21-23). These relationships are in many cases explainable by hormonal or autonomic systems mechanisms based on the conventional medicine. Therefore in uterine strangulation the neuropsychiatric signs/symptoms occur due to the uterus problem (21-23). The importance of correct diagnosis of these patients is due to the treatment focus should be on uterus instead of the brain or psychiatric therapies (21). As mentioned before, two groups of women are predispose to uterine strangulation; one is those with oligomenorrhea, amenorrhea or hypomenorrhea and the other those in the fertility ages that do not have sexual relationship. The first group has better prognosis and response to treatment (21-23).

**Signs/** **symptoms**

Because of the relationship between body organs which is well explained in the ITM textbooks, although the uterine strangulation originates from the uterus, it shows systemic signs and symptoms (19-21). These are classified according to different involved systems of the body as shown in table I (22). Based on ITM humoral theories, the symptoms and signs may differ in accordance to the dominant humor that caused the disease (21). In table II symptoms are listed based on the causal dominant humor (21). Moreover it is important to note that uterine strangulation is a periodical disease which exacerbates in the autumn.

**Table I:** Uterine strangulation syndrome signs and symptoms

**Cardiovascular symptoms**	**Respiratory symptoms**	**Psychiatric** **symptoms**	**Neurologic symptoms**	**Systemic symptoms**
Syncope	Breathing problems	Anxiety	involuntary movements in face and lips	halitosis
Bradycardia	Dyspnea	Hallucination	dizziness	anorexia
Faint		Delusion	headache	asthenia feeling in leg
Palpitation			speaking disorder	changing the face color to yellow or black
			sensory/motor weakness	

**Table II:** Semiology based on the humoral etiology of the uterine strangulation

**Biliary humor**	**Phlegmatic humor**	**Melancholic humor**
better prognosis	feeling of asthenia	the worste form of the disease
more severe signs and symptoms	symptoms are less than melancholic-based disease	severe faint
		obsessive-compulsive disorder
		eye pain
		nausia and vomitting
		problem in breathing

**Treatments**

During the attack, though massage of the sole and special aromatherapy techniques are recommended. At the time between attacks for causal-based treatment, if the menstrual bleeding cessation is the underlying mechanism for the uterine strangulation syndrome, uterine cleansing techniques (stave off the excreta) and inducing the menstrual bleeding are the main treatment strategies. Several methods are applied in the ITM for this purpose including herbal therapy, dry cupping (sites: under the umbilicus, knees and leg) and other methods. These treatments are all prescribed in accordance to the basic (and also the uterine) temperament of the patient (19-21).

**Conventional** **(western) medicine evidences on uterine strangulation**

The authors have searched available scientific data banks and journals including PubMed and Google Scholar with wide range of keywords including oligomenorrhea, menstrual bleeding cessation, menstruation, menstrual, psychotic, hysteria, epilepsy, headache, amenorrhea, menstruation, emotional, PCOD, polycystic ovarian syndrome (PCOS), polycystic ovarian disease, etc; however they could not find any exact equivalent syndrome for the uterine strangulation in the conventional medicine; Although many researchers have evaluated and confirmed the relationship between changes in the menstrual cycle and mood/ mental state/ psychiatric symptoms (24-37). It is confirmed that there is a significant risk for mood disorders including anxiety, depression, personality disorders and bipolar disorders in women with PCOS (32, 35). 

Reviewing the literature, the authors realized that a condition which seems comparable to uterine strangulation attacks is epilepsy/seizure. Several studies have demonstrated that reproductive endocrine disorders in particular PCOD, is increased in epilepsy, independent of antiepileptic medications or type of seizure disorder (38-42). Herzog *et al* reported a 20% occurrence of PCOD in a group of 50 women with temporal lobe epilepsy (39). However the ITM textbooks point out the following differences between uterine strangulation and seizure attacks: In an attack of uterine strangulation, mouth foaming and tongue biting is unusual, also the patient remembers all phases of the attack in the uterine strangulation in contrast to seizure. Consequently, it seems that pseudo-seizure or hysteria is more familiar to this syndrome, although the etiology and physiopathology mentioned in ITM textbooks are never proposed in the conventional medicine under the title of pseudo-seizure or hysteria. 

Patients who suffer from uterine strangulation syndrome are referred to neurologists and psychiatrists every day without having a clear diagnosis or receiving an effective treatment; since the collection of such symptoms and signs is not mentioned in the conventional medicine resources. For example Yutzy *et al* reported the following case with several systemic symptoms with no definite diagnosis; we discern that this case may suffer from uterine strangulation according to ITM knowledge (43).

‘A 35-year-old woman presented with a complaint of extreme headaches, "like a knife being stuck through the back of my head into my eye," as well as other headaches virtually every day. After medical and neurological examinations failed to suggest any specific etiology for either headache, it was important to take a careful history of past symptoms. In this case, the woman also reported a history of other pains, including abdominal pain associated at times with nausea and vomiting, periods of constipation followed by diarrhea which had resulted in investigation for gallbladder and peptic ulcer disease with no significant findings, and pain "in all of my joints" but particularly in her knees and her back that she said had been diagnosed as degenerative arthritis at age 27 years yet no deformities had developed since. She had had menstrual problems since menarche, with pain that put her to bed and excessive flow with "big blue clots", which had resolved only after hysterectomy two years earlier at age 33 years. 

The mother of four, she reported a long history of sexual problems including pain with intercourse. She had been told that she has a "tipped uterus". Throughout her life, she was seldom orgasmic and had not enjoyed sex "for years". She reported episodes of blurred vision with "spots" in front of her eyes, which caused her to stop work, and other episodes when she just could not hear anything, "like someone put their hands over my ears." She also reported periods of uncontrollable shaking and a feeling that she was losing control of her body, for which she had been investigated for seizures. She reported that, at times, she had feared having some serious medical disease but "with all the work-ups I have had, I am sure they would have found something by now" (43).

Some of the patients with uterine strangulation (in the type with lack of sexual satisfaction etiology) may be classified in to the somatization disorder. The DSM-IV criteria for Somatization disorder is as follow: at least four pain symptoms [headaches, abdominal pain, back pain and knee pain], at least two non-pain gastrointestinal symptoms [nausea, vomiting, diarrhea and constipation], at least one sexual or reproductive symptom [pain on intercourse, excessive menstrual flow, loss of sexual enjoyment] and at least one pseudoneurological symptom [muffled hearing, uncontrollable shaking, blurred vision, spots in visual field] (44). However the patient treatment protocol for somatization disorder is not defined on the basis of menstrual bleeding or sexual satisfaction therapy in the conventional medicine. 

## Conclusion

The high and increasing prevalence of oligomenorrhea causes a lot of patients refer to the ITM clinics with this complaint daily (1, 6, 8). Uterine strangulation is one of the most common features of oligomenorrhea according to ITM textbooks which has not been cited in the conventional medicine. The authors have searched available resources for the possible evidences in the conventional medicine literature for this syndrome. This review showed that there is a significant risk for mood and psychiatric disorders in women with PCOS (as the most prominent group suffering from oligomenorrhea) (32, 35).

Several studies have shown that women with PCOS are more likely to suffer from anxiety symptoms (45, 46). Benson *et al *showed that 34% of women with PCOS have clinically significant anxiety (47). Manson *et al* found that as well as the generalized anxiety disorders; social phobia is increased in PCOS women (48). Sahingöz *et al* found a prevalence rate of any personality disorder of 23.3% in the women with PCOS and 9.6% in the control group (49). In a meta-analysis by Dokras *et al* social phobia, specific phobia, panic disorder and depression were found to be more common in patients with PCOS (50). Likewise, in Harmanci *et al* study, increased phobic disorder and obsessive compulsive symptoms were revealed comparing to the control group (51).

Few other studies have shown some relations with interpersonal sensitivity, social anxiety, aggression, suicidal behavior, and more suspicious personality traits compared to healthy controls in PCOS group (52, 53) Unfortunately, these studies mostly focused on the symptoms rather than on the syndromal psychiatric disorders (46, 47, 54-57). The causal relationship between PCOS and psychiatric disorders is unknown (49). Some causal parameters for the higher prevalence of psychotic symptoms in PCOS patients may include the Physical symptoms associated with PCOS (such as hirsutism, obesity, cystic acne, seborrhea and hair loss) and the higher rate of infertility in this group (54-58). On the other hand, insulin resistance and a higher body mass index (BMI) in women have been reported to be related to clinical mood disorders in women with PCOS (59). Moreover, augmented levels of androgens in women have been shown to be related to mood disorders like depressive mood, irritability and aggression in women with PCOS (60-63).

Some of the uterine strangulation symptoms like dyspnea, palpitation, mental disorders, anxiety, speech problems and breathing problems are in accordance with the results from these researches; it seems that anxiety plays an important role in uterine strangulation syndrome. Some other symptoms which can be classified under the sensory/motor category are mostly familiar with seizure/pseudoseizure signs. Several studies have demonstrated some relationship between epilepsy and PCOD, independent of antiepileptic medications or type of seizure disorder (38-42).

In conclusion several cases of oligomenorrhea with systemic symptoms have been treated successfully in ITM clinics with the diagnosis of uterine strangulation during the past years. The treatment protocol is based on induction of menstrual bleeding to expel the excreta material from the body (19-21). Approving this hypothesis on physiopathology and treatment of uterine strangulation, needs evidence- based strong studies. Patients with uterine strangulation symptoms, who are referred undecided between neurologists, psychiatrists and cardiologists, would gain the most from these studies.

## Conflict of interest

The authors declare that there is no conflict of interests regarding the publication of this article.
